# Detecting fatigue in multiple sclerosis through automatic speech analysis

**DOI:** 10.3389/fnhum.2024.1449388

**Published:** 2024-09-13

**Authors:** Marcelo Dias, Felix Dörr, Susett Garthof, Simona Schäfer, Julia Elmers, Louisa Schwed, Nicklas Linz, James Overell, Helen Hayward-Koennecke, Johannes Tröger, Alexandra König, Anja Dillenseger, Björn Tackenberg, Tjalf Ziemssen

**Affiliations:** ^1^ki:elements GmbH, Saarbrücken, Germany; ^2^Center of Clinical Neuroscience, Department of Neurology, University Clinic Carl Gustav Carus Dresden, TU Dresden, Dresden, Germany; ^3^F. Hoffmann La Roche AG, Basel, Switzerland; ^4^^Department of Clinical Neuroscience^, University of Glasgow, Glasgow, United Kingdom; ^5^University of Côte d’Azure, Nice, France; ^6^Department of Neurology, Philipps University, Marburg, Germany

**Keywords:** multiple sclerosis (MS), fatigue, speech, automated speech analysis, machine learning

## Abstract

Multiple sclerosis (MS) is a chronic neuroinflammatory disease characterized by central nervous system demyelination and axonal degeneration. Fatigue affects a major portion of MS patients, significantly impairing their daily activities and quality of life. Despite its prevalence, the mechanisms underlying fatigue in MS are poorly understood, and measuring fatigue remains a challenging task. This study evaluates the efficacy of automated speech analysis in detecting fatigue in MS patients. MS patients underwent a detailed clinical assessment and performed a comprehensive speech protocol. Using features from three different free speech tasks and a proprietary cognition score, our support vector machine model achieved an AUC on the ROC of 0.74 in detecting fatigue. Using only free speech features evoked from a picture description task we obtained an AUC of 0.68. This indicates that specific free speech patterns can be useful in detecting fatigue. Moreover, cognitive fatigue was significantly associated with lower speech ratio in free speech (*ρ* = −0.283, *p* = 0.001), suggesting that it may represent a specific marker of fatigue in MS patients. Together, our results show that automated speech analysis, of a single narrative free speech task, offers an objective, ecologically valid and low-burden method for fatigue assessment. Speech analysis tools offer promising potential applications in clinical practice for improving disease monitoring and management.

## 1 Introduction

MS is a chronic neuroinflammatory disease characterized by demyelination and axonal degeneration in the central nervous system (CNS) (Soler et al., 2020) causing a variety of symptoms, depending on the location of the lesions. One of the most common symptoms is fatigue (up to 81% of MS patients are affected) (Kister et al., 2013), which describes a state of extreme tiredness and lack of energy. Fatigue significantly impairs the daily activities of those affected (Krupp et al., 1988). For many patients, fatigue is considered the most debilitating symptom, impacting quality of life more than physical disability and pain (Janardhan and Bakshi, 2002). Due to its widespread impact on daily activities, fatigue leads to significant socioeconomic consequences, affecting employment status, capacity to work, and frequency of sick leaves (Oliva Ramirez et al., 2021). In addition to its personal impact, the socioeconomic implications of fatigue in people with MS (pwMS) make it a critical target for treatment. Effectively addressing and mitigating fatigue in MS is essential for improving patient outcomes and reducing the broader social and economic burden associated with the disease.

Current treatments for MS can reduce clinical relapses and new lesion formation (De Angelis et al., 2018), but they do not reverse existing tissue damage or effectively control chronic symptoms such as fatigue, which persist across different types of MS (Herring et al., 2021). Despite its prevalence and impact, the underlying mechanisms of fatigue in MS are poorly understood. Recent research suggests that fatigue in MS likely has multiple causes, including immune activation and the release of proinflammatory cytokines, chronic CNS damage from lesions and axonal loss, altered brain activity patterns due to tissue loss, and altered endocrine function (Braley and Chervin, 2010). Secondary factors, such as depression and sleep disturbances, also contribute to fatigue in MS (Bhattarai et al., 2023; Ormstad et al., 2020). Accurate measurement of fatigue is crucial for the development of targeted treatments, as it allows for precise identification of contributing factors, assessment of treatment efficacy, and the tailoring of interventions to address the specific needs and conditions of individual patients (Pinarello et al., 2023).

Despite recent developments, current fatigue assessment tools and strategies face several limitations, primarily concerning their ability to accurately capture the multifaceted nature of fatigue. Many tools rely heavily on patient-reported outcomes, which are susceptible to poor content validity, leading to type-II errors and under-detection of fatigue (Close et al., 2023). Additionally, there is a lack of standardized measures across different conditions and populations, complicating the comparison of results and the generalization of findings (Whitehead, 2009). Objective measures, such as actigraphy, are limited by their inability to differentiate between physical and mental fatigue, and fatigability, thus failing to provide a comprehensive assessment (Gulde and Rieckmann, 2022). Moreover, comorbidities such as sleep disorders complicate the accurate assessment of fatigue in pwMS (Paucke et al., 2018). Together with its heterogeneous etiology, the lack of a unified definition and the subjective nature of the fatigue, influenced by various psychological, social, and environmental factors, poses challenges in developing universally applicable and sensitive assessment tools.

Given the significant negative impact of fatigue on the quality of life in pwMS, recent efforts have focused on developing more accurate methods to detect fatigue, with the goal of facilitating its treatment (Pinarello et al., 2023). Automated speech analysis has emerged as a potential low-burden and non-invasive method for detecting fatigue in pwMS. Speech analysis methods have been effective in detecting fatigue in other contexts, such as COVID-19 patients, sleep deprivation, air traffic controllers and aviation pilots (De Vasconcelos et al., 2019; Elbéji et al., 2022; Gao et al., 2022; Greeley et al., 2007; Vogel et al., 2010; Xu et al., 2023). Interestingly, in the context of MS, patients report greater verbal communication impairments due to fatigue (Hartelius et al., 2004), and the onset of these deficits is often associated with fatigue onset (Blaney and Lowe-Strong, 2009). These findings support the hypothesis that verbal communication, and speech in particular, may be significantly associated with fatigue symptoms in pwMS. This suggests that automated speech analysis tools could be used to detect fatigue.

Therefore, this study aims to evaluate the efficacy of automated speech analysis in identifying fatigue among pwMS, thereby contributing to improved disease monitoring and management strategies.

## 2 Materials and methods

### 2.1 Participants

A total of 297 subjects (Table 1) participated in this study, including 142 MS patients who received treatment at the MS Center Dresden (Germany) and 155 healthy controls. The control group was recruited via posters placed in the same center. All participants were 18 years or older, native German speakers and provided written informed consent to participate in the study according to the Helsinki declaration (World Medical Association, 2013). Furthermore, the study was approved by the local ethics board of the MS Center Dresden.

**Table 1:** Demographics overview of the final dataset after pre-processing.

	Total	HC	pwMS	*p*-value
*n*	297	155	142	
Gender	F: 205; M: 92	F: 104; M: 51	F: 101; M: 41	
Age (years)	41.74 (13.55)	39.7 (14.99)	43.97 (11.41)	< 0.001
Education (years)	13.44 (2.81)	13.38 (2.64)	13.51 (2.99)	0.80
EDSS	2.98 (1.52)		2.98 (1.52)	
EDSS Severity subgroups	0: 155 ≤ 3: 96 ≤ 7: 43 ≤ 10: 3	0: 155	0: 0 ≤ 3: 96 ≤ 7: 43 ≤ 10: 3	
MS Subtype			RRMS: 119 PPMS: 11 SPMS: 10 CNS: 1 unknown: 1	
FSMC	44.03 (20.95)	32.03 (10.67)	57.13 (21.59)	< 0.001
FSMC motor	22.24 (11.09)	15.93 (5.3)	29.13 (11.66)	< 0.001
FSMC cognition	22.24 (10.77)	16.29 (6.07)	28.73 (11.04)	< 0.001
HADS-Anxiety	4.94 (3.64)	4.06 (2.9)	5.9 (4.1)	< 0.001
HADS-Depression	3.29 (3.64),	2.04 (2.42)	4.65 (4.22)	< 0.001
SDMT (number of correctly solved items)	23.74 (8.15)		23.74 (8.15)	
9-HPT dominant hand (seconds)	23.48 (6.92)		23.48 (6.92)	
9-HPT non-dominant hand (seconds)	23.48 (6.92)		23.48 (6.92)	
T25-FW	5.04 (2.71)		5.04 (2.71)	

For age, education and EDSS, FSMC, SDMT and 9-HPT, the mean (standard deviation) is reported. Group differences were computed using Kruskal-Wallis test. FSMC, Fatigue Scale for Motor and Cognition. HADS, Hospital Anxiety and Depression Scale. SDMT, Symbol Digit Modalities Test. 9-HPT, Nine Hole Peg Test. T25-FW, Timed 25 Foot Walking Test. RRMS, Relapsing–Remitting MS Subtype. PPMS, Primary Progressive MS Subtype. SPMS, Secondary Progressive MS Subtype. CNS, Chronic inflammatory disease of the central nervous system.

### 2.2 Assessments and speech tasks

The assessments were performed in a fixed order with a pause between each scale/test. The entire assessment battery consisted of tests and questionnaires, as well as general clinical routine assessments for cognitive ability, including the Expanded Disability Status Scale (EDSS; Kurtzke, 1983), the Nine-Hole Peg Test (9HPT, Feys et al., 2017), the Timed 25 Foot Walk Test (T25-FW; Motl et al., 2017), the Symbol Digit Modalities Test (SDTM; Benedict et al., 2017), the Hospital Anxiety and Depression Scale (HADS; Spinhoven et al., 1997), the Quality of Life in Neurological Disorders questionnaire (Neuro-QoL; Cella et al., 2012) and the Fatigue Scale for Motor & Cognition (FSMC; Penner et al., 2009).

Moreover, all participants from both groups conducted a speech assessment protocol consisting of nine tasks, testing the articulatory, phonatory and narrative dimensions of speech. The speech assessment protocol was conducted on a tablet-based app, Mili, developed and maintained by ki elements GmbH.^1^ The participants’ voice was recorded using the tablet’s internal microphones and stored online on the ki:elements’ server located on the premises of the University Clinic Dresden. The full speech protocol consisted of the picture description task, a narrative storytelling task, where participants recalled a positive and a negative personal episode, a semantic verbal fluency task, a sustained phonation task (vowel ‘a’), an articulatory task (successively repeating the Pa-Ta-Ka syllables), the California Verbal Learning Test (CVLT; Delis et al., 1988) and the logical memory task of the Wechsler Memory Scale (LM-WMS; Wechsler, 2009). With the goal of maximizing ecological validity, in this study, we only focused on the free narrative speech tasks (i.e.: positive and negative storytelling, and picture description).

All assessments were conducted in German. The speech recordings were performed in a quiet room at the MS Center Dresden under the supervision of a speech scientist. Efforts were made to maintain environmental conditions as consistent as possible across all participants.

### 2.3 Data analysis

Before extraction of speech features, all sound files were preprocessed. The initial step involved excluding participants who did not complete all the speech assessments or had at least one audio file shorter than 5 s (*N* = 25). Next, acoustic and linguistic features were extracted from the speech tasks. Acoustic features were extracted directly from the audio recordings while linguistic features were computed on transcripts that were automatically generated using google speech-to-text Automatic Speech Recognition (ASR) services. Transcripts were quality checked by listening to randomly selected recordings and comparing them to ASR transcripts. Then, features were extracted using the ki:elements speech processing library SIGMA 14.0.0. Additionally, based on features extracted from the semantic verbal fluency task and the CVLT, the ki:elements speech biomarker for cognition (SB-C) was computed.

The features of the free speech tasks (positive and negative storytelling, and picture description) in conjunction with the SB-C and its subscores were used to train a support vector machine (SVM) classifier to discriminate between fatigued (FSMC total score ≥ 43) and non-fatigued (FSMC total score < 43) participants. From a total of 362 features, we sorted all features based on mutual information and tested performance in 10-fold cross validation on a 10% left-out test set. Reported performance metrics are means over the 10 iterations. We varied *k* between 10 and 250. Then, we compared the performance of the different classifiers using balanced accuracy and area under the receiver operator curve (AUC) as a function of *k* to obtain the best tradeoff between explainability and performance. The trade-off was determined by visually identifying the point of inflection of performance as a function of *k* features. The number of features (*k*) was selected based on this visual inspection.

In the next step, to increase the explainability of our results, we selected the *k* features used for classification and computed their intercorrelations and their correlations with the FSMC (Penner et al., 2009) to assess the association between speech features and fatigue. Given the non-normal distribution of some features and the potential non-linear relationships, we employed non-parametric Spearman Rank-Sum correlations. This approach allowed us to better understand the connections between specific speech characteristics and the different dimensions of fatigue in MS patients.

Finally, to better understand the relationship between fatigue and other relevant symptoms we used Spearman Rank-Sum correlations to test the association of fatigue and established neuropsychological tests and their sub-scores, including the EDSS (Kurtzke, 1983), 9HPT (Feys et al., 2017), T25-FW (Motl et al., 2017), the SDTM (Benedict et al., 2017) and the HADS (Spinhoven et al., 1997). In testing these correlations, we controlled for general disability by using EDSS scores.

## 3 Results

### 3.1 Free speech features accurately characterize fatigue

**Figure 1:**
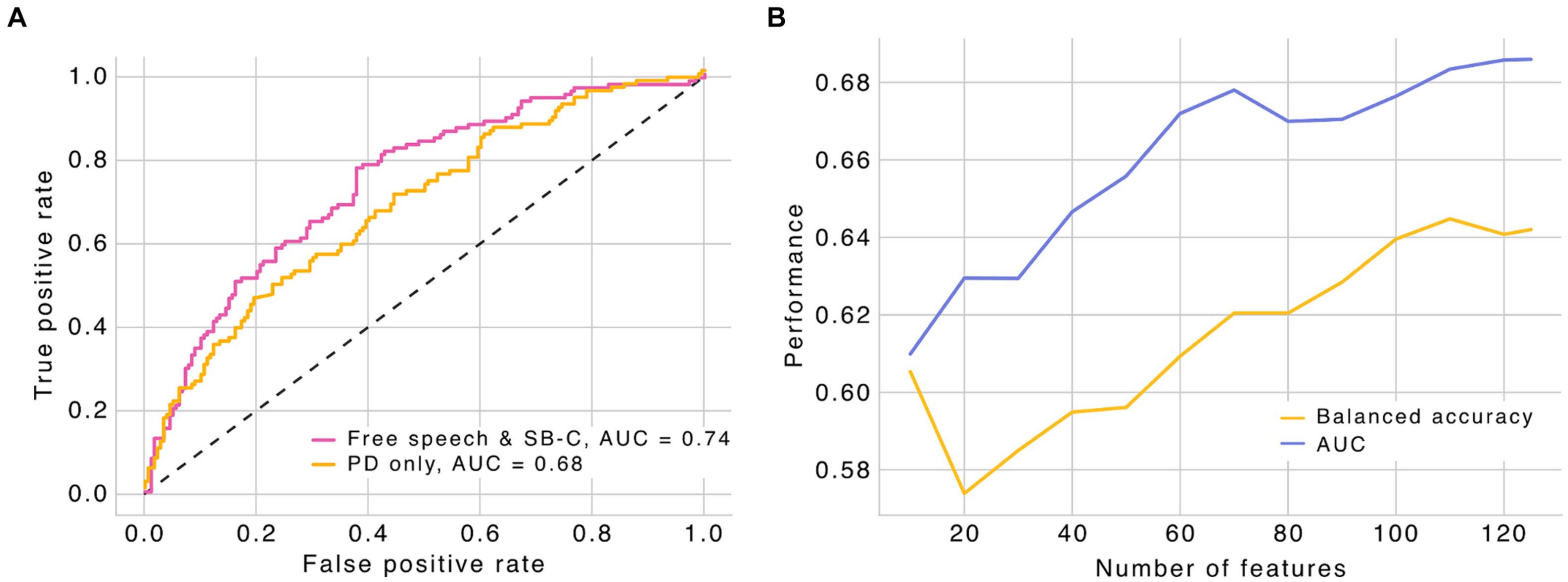
**(A)** Receiver operating characteristic curve (ROC) for the different SVM models. Free speech includes negative and positive storytelling, and picture description. Performance metrics were computed based on a 10-fold cross validation strategy with a 90–10% train-test splits. Black dashed line represents the chance level. **(B)** Performance metrics for the picture description model as a function of number of features. PD, picture description; SB-C, speech biomarker for cognition (ki:elements). AUC, area under the curve.

To test the feasibility of using automated speech analysis to detect fatigue in pwMS, we focused our analysis on free speech tasks (positive and negative storytelling, and picture description) applied to a group of MS patients and a group of healthy controls (Table 1). In our sample, pwMS were, on average, approximately 4 years older than controls. Moroever, as expected (Walton et al., 2020), women were more prevalent in the MS group. This gender imbalance was consistent between groups. We employed support vector machine (SVM) models to predict the presence of fatigue in both pwMS and control subjects. To maximize fatigue detection, we started by testing a model that included all free speech tasks along with our proprietary cognition score derived from speech (SB-C), given the potential association between fatigue and cognitive symptoms (Guillemin et al., 2022). For this more complex model, we observed an area under the curve (AUC) of 0.74 on the receiver operating characteristic (ROC) curve when using the 150 most relevant features identified through feature selection (balanced accuracy = 0.67). This model performed significantly above chance level (Figure 1A; AUC = 0.5, balanced accuracy = 0.5), suggesting that free speech contains relevant information and that features extracted from it may be relevant for fatigue detection.

Building on these findings, we further explored whether a simplified approach, focusing on the most relevant speech features from the picture description task alone, could maintain high predictive accuracy for fatigue detection. We observed that a simplified model, which includes only speech features from the picture description task, yielded comparable results with minimal loss of performance (Figure 1A), suggesting that the picture description task can be useful in predicting fatigue in pwMS. Specifically, using speech features extracted exclusively from the picture description task, we found an AUC of 0.68 for the ROC curve with only 70 features (balanced accuracy = 0.62), the number of features where the model reaches stable levels of performance. Adding more features only marginally improved the model performance (Figure 1B), suggesting that a subset of features derived from free speech may be sufficient to detect fatigue. When carefully analyzing the features selected based on mutual information (i.e., the 27 features selected in all 10 folds of the model cross validation; Supplementary Table 2), we found that a significant portion relate to speech structure (e.g., pause duration and speech rate) and voice quality (e.g., pitch-related features), highlighting the importance of tracking these features during free speech for detecting fatigue.

Together, our modeling data indicate that speech features extracted from the picture description task are sufficient to predict fatigue above chance level, highlighting the utility of free speech as an effective tool for revealing fatigue-related alterations.

### 3.2 Speech ratio significantly correlates with cognitive fatigue

Having established that features extracted from free speech are relevant for fatigue detection, we sought to better understand the relevance of key speech features. To achieve this, we correlated fatigue scores (cognitive, motor, and total FSMC scores) exclusively with the 27 features that were common across all instances of the model’s cross-validation (10 folds). Using this unbiased strategy, we computed correlations between fatigue and 27 speech features derived from the picture description task (Supplementary Table 1).

Our results show that the overall fatigue score positively correlates with pause rate (adjusted *p* = 0.036), mean pause duration (adjusted *p* < 0.001) and its standard deviation (adjusted *p* < 0.001), and the standard deviation of the F1 relative energy (relative energy between the fundamental frequency [F0] and the first formant [F1]) (adjusted *p* = 0.007). Moreover, the total fatigue score negatively correlates with speech ratio (adjusted *p* < 0.001), adjective rate (adjusted *p* = 0.007) and Brunet’s index (*p* = 0.022) (Table 2).

**Table 2:** Significant correlations, based on adjusted *p*-values, between fatigue and picture description features.

	Coefficient	*p*-value	Effect size	Adjusted *p*-value
	FSMC - total score			
Mean pause duration	0.263	0.0	0.545	0.0
Pause durations (SD)	0.234	0.0	0.482	0.0
Speech ratio	−0.237	0.0	−0.488	0.0
F1 relative energy (SD)	0.188	0.001	0.382	0.007
Adjective rate	−0.184	0.002	−0.374	0.007
Brunet’s index*	−0.161	0.005	−0.327	0.022
Pause rate	0.149	0.010	0.301	0.036
	FSMC - motor score			
Mean pause duration	0.248	0.0	0.512	0.0
Pause durations (SD)	0.232	0.0	0.478	0.001
Speech ratio	−0.219	0.0	−0.450	0.001
F1 relative energy (SD)	0.199	0.001	0.407	0.004
Adjective rate	−0.171	0.003	−0.346	0.015
	FSMC - cognitive score			
Mean pause duration	0.242	0.0	0.500	0.001
Speech ratio	−0.234	0.0	−0.482	0.001
Pause durations (SD)	0.199	0.001	0.407	0.005
Brunet’s index*	−0.171	0.003	−0.348	0.015
Adjective rate	−0.174	0.003	−0.354	0.015
F1 relative energy (SD)	0.153	0.008	0.311	0.029
Number of pauses	−0.155	0.008	−0.315	0.029

FSMC, Fatigue Scale for Motor and Cognition; SD, standard deviation; F1, first formant *Brunet’s index; a measure of lexical diversity.

When analyzing motor fatigue scores (Table 2), we found positive correlations with mean pause duration (adjusted *p* < 0.001) and its standard deviation (adjusted *p* = 0.001), and the standard deviation of the F1 relative energy (adjusted *p* = 0.004). Additionally, we found that motor fatigue negatively correlates with speech ratio (adjusted *p* = 0.001) and adjective rate (adjusted *p* = 0.015).

For cognitive scores (Table 2), mean pause duration (adjusted *p* = 0.001), its standard deviation (adjusted *p* = 0.005) and the standard deviation of the F1 relative energy (adjusted *p* = 0.029) positively correlate with cognitive fatigue. Finally, speech ratio (adjusted *p* = 0.001), number of pauses (*p* = 0.029), adjective rate (adjusted *p* = 0.015) and Brunet’s index (*p* = 0.015) negatively correlate with cognitive fatigue.

**Figure 2:**
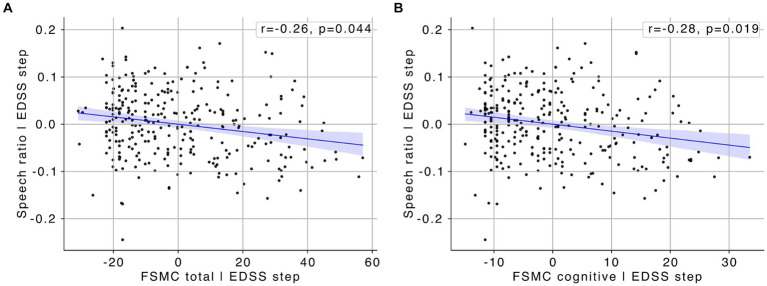
**(A)** Scatter plot showing the partial correlation between FSMC total scores and speech ratio, with the effect of EDSS scores statistically controlled. **(B)** Scatter plot showing the partial correlation between FSMC cognitive scores and speech ratio, with the effect of EDSS scores statistically controlled. Blue lines represent the line of best fit, with the shaded areas indicating the 95% confidence interval.

However, when controlling for EDSS score, only the negative correlations of speech ratio with cognitive fatigue (*ρ* = −0.283; *p* = 0.001; effect size = −0.590; adjusted *p* = 0.019) and overall fatigue (*ρ* = −0.264; *p* = 0.002; effect size = −0.547; adjusted *p* = 0.044) remained significant (Figures 2A,B). This suggests that, although moderate, the relationship between speech ratio and fatigue is independent of overall disability level, indicating that speech ratio might be a specific marker of fatigue, particularly cognitive fatigue, rather than being broadly associated with disability severity in individuals with MS.

### 3.3 Fatigue is associated with other symptoms

To better understand the association between clinical symptoms and fatigue, we performed a correlation analysis between results of all scales and tests, and the FSMC scores. To account for general disability level, we computed partial correlations, partialling out the effect of the EDSS score. The analysis revealed that fatigue (FSMC total score) was significantly associated with a number of clinically relevant symptoms, including mood (*ρ* = 0.555; *p* < 0.001; effect size = 1.335; adjusted *p* < 0.001), anxiety (*ρ* = 0.542; p < 0.001; effect size = 1.289; adjusted *p* < 0.001), lower (*ρ* = −0.395; *p* < 0.001; effect size = −0.860; adjusted *p* < 0.001) and upper extremity function (*ρ* = −0.478; *p* < 0.001; effect size = −1.087; adjusted *p* < 0.001), and cognition (Neuro-Qol questionnaire; *ρ* = −0.663; *p* < 0.001; effect size = −1.769; adjusted *p* < 0.001) (see Supplementary Table 1 for full list of correlations). However, we did not find significant associations between fatigue and processing speed (*ρ* = −0.085; *p* = 0.315; effect size = −0.171; adjusted *p* = 0.333) or memory function (*ρ* = −0.099; *p* = 0.243; effect size = −0.199; adjusted *p* = 0.272), two domains known to be affected in MS (Chiaravalloti and DeLuca, 2008; Langdon, 2011).

Although our analyses do not allow us to disentangle the causal direction of effects, the associations we found highlight the multifaceted nature of fatigue in MS and its extensive impact on various aspects of patient well-being. Using speech analysis to assess fatigue over extended periods of time (i.e., longitudinally) may offer additional insights into the complex interactions between clinical symptoms and fatigue in MS.

## 4 Discussion

Fatigue is a pervasive symptom in pwMS, significantly impacting their quality of life and daily functioning (Janardhan and Bakshi, 2002; Krupp et al., 1988). As a multidimensional construct, fatigue is commonly assessed using questionnaires that include various subscales targeting different dimensions of fatigue (Chalder et al., 1993; Krupp, 1989; Penner et al., 2009; Piper et al., 1998; Smets et al., 1995). However, these traditional methods often rely on subjective self-reports, which can be influenced by various factors and lack standardization (Close et al., 2023). Moreover, fatigue presents heterogeneously, is highly subjective, and lacks a unified definition, making it challenging to measure accurately. Therefore, developing new tools and methods to precisely measure fatigue is paramount for advancing treatment strategies for fatigue in MS. In this work, we provide evidence on the feasibility of using automated analysis tools to detect fatigue from free speech.

Free narrative speech has proven useful in identifying disabilities associated with various neurological disorders (Fraser et al., 2016; König et al., 2015; Lindsay et al., 2019; Mefford et al., 2023) and psychiatric diseases (Alpert et al., 2001; Bedi et al., 2015), including in MS (Svindt et al., 2020, 2023). Free speech tasks, such as the picture description task, elicit spontaneous and natural speech patterns that are more reflective of everyday communication. This ecological validity is crucial for accurately assessing the impact of neurological conditions on speech and cognition. Furthermore, free speech tasks can capture a wide range of linguistic and paralinguistic features, such as speech rate, fluency, lexical diversity, and voice quality, which are often affected in these disorders (Fraser et al., 2016; König et al., 2015). However, speech protocols tend to be composed of different tasks, targeting different dimensions of speech. Despite their richness, conducting such an extensive speech protocol is time-consuming and cognitively demanding, particularly for populations reporting significant fatigue, such as pwMS. Additionally, the process of extracting and analyzing speech features is both lengthy and complex, highlighting the need for simplified assessments.

In this study, we tested the feasibility of using a single free speech task to predict fatigue, focusing on speech features extracted from a picture description task. This task has been shown to accurately capture relevant cognitive and linguistic markers in different diseases, including Amyotrophic Lateral Sclerosis, mild congitive impaiment and Alzheimer’s disease (e.g., Lindsay et al., 2019; Mueller et al., 2018), which may be associated with fatigue (Wallace and Holmes, 1993). The picture description task involves describing a complex image, which provides a rich source of linguistic data that can be analyzed for features such as speech rate, fluency, and lexical diversity, all of which have been correlated with cognitive changes (Cho et al., 2021; Henderson et al., 2023; Skodda and Schlegel, 2008). This task is relatively simple to implement and represents a low burden to patients. By concentrating on this task, we aimed to streamline the assessment process while maintaining accuracy in fatigue detection.

We observed that 70 free speech features, prompted by a picture description task, provide significant information for detecting fatigue in pwMS. Notably, our model achieved high accuracy levels, comparable to a more complex model that included three tasks and 150 features. This finding suggests that focusing on free narrative speech as an assessment tool may be sufficient, thereby avoiding lengthy assessment protocols and the complexity of analyzing extensive datasets. Moreover, free narrative speech assessments could potentially be conducted during face-to-face or video call appointments between patients and medical care providers. Additionally, we found that most of these features relate to speech structure, semantic richness, and voice quality, indicating their robustness as indicators of fatigue. This aligns with previous research showing that speech patterns change in response to fatigue and that they may reflect underlying cognitive and motor impairments (Blaney and Lowe-Strong, 2009; Hartelius et al., 2004).

Altough moderate, our correlation results further underscore the relevance of specific speech features. For instance, mean pause duration positively correlated with overall and cognitive fatigue, while speech ratio and adjective rate negatively correlated with these fatigue measures. Interestingly, when controlling for EDSS score, the negative correlations between speech ratio and both cognitive and overall fatigue remained significant. This suggests that certain speech alterations may be mediated by overall disability and that speech ratio may serve as a robust specific marker of fatigue independent of general disability level. However, the moderate correlation coefficient indicates that a substantial portion of variability cannot be accounted for by the linear relationship between fatigue and speech ratio. Moreover, it indicates that other factors, besides fatigue, may significantly contribute to speech ratio variability, suggesting that further studies are needed to fully understand this association. Although this relationship is not exceedingly strong, it indicates that fatigue has a noticeable impact on speech production, warranting further investigation and consideration in clinical assessments.

One limitation of our study is that it exclusively focused on German-speaking participants, potentially limiting the applicability of the findings to other linguistic and cultural contexts. Speech characteristics can vary significantly across languages, which may influence the effectiveness of automated speech analysis tools. This emphasizes the need for cross-linguistic validation of diagnostic tools (Lau et al., 2022; Parola et al., 2022). Another relevant limitation pertains to the gender of participants. As in other MS studies, our sample predominantly consisted of female participants. MS is known to affect females more frequently than males, but the overrepresentation of females in our study may limit the generalizability of the findings to the male MS population. Speech characteristics and fatigue manifestations may differ between genders, thus necessitating further studies with a more balanced gender distribution to ensure that the diagnostic tools are equally effective for both males and females. Additionally, the cross-sectional design of the study restricts the ability to infer causality between speech changes and fatigue. Moreover, fatigue fluctuates significantly within and between days and may have been suboptimally detected on the day of the assessment, particulalry in the case of the relapsing–remitting form of MS (Powell et al., 2017). Longitudinal studies are necessary to establish the temporal relationships and provide a more comprehensive understanding of symptom dynamics over time. This highlights the need for continuous monitoring to understand and address the progression of symptoms effectively. We propose a longitudinal follow-up study where fatigue and speech assessments are performed regularly, over an extended peiord of time, in hopes of overcomming this limitaiton and confirming our findings.

Automated speech analysis offers several advantages over traditional fatigue assessment tools. It provides an objective measure that is less susceptible to biases inherent in self-reported data. Additionally, it can capture subtle changes in speech that may not be evident through subjective assessments. This method can be easily integrated into routine clinical practice, or even through remote assessments, providing continuous monitoring of fatigue and enabling timely interventions. The use of automated speech analysis in MS could help improve fatigue assessment, offering a low-burden, scalable, and precise tool for clinicians and researchers. Future studies should explore the longitudinal application of this method and its integration with other biomarkers to enhance the understanding and management of fatigue in MS. Furthermore, the development of standardized protocols for speech tasks and feature extraction will be crucial to ensure the reliability and generalizability of findings across different populations and settings. Extending these methods to cross-cultural studies will help validate and refine the approach for broader application.

In conclusion, this study demonstrates that automated speech analysis, particularly through a single narrative free speech task, provides an effective and low-burden method for detecting fatigue in multiple sclerosis patients. The findings highlight the potential of integrating speech analysis tools into clinical practice for improved monitoring and management of fatigue in MS.

## Data Availability

The datasets presented in this article are not readily available because of commercial conflict. Requests to access the datasets should be directed to Nicklas Linz, nicklas.linz@ki-elements.de.

## Footnotes

^1^https://ki-elements.de
