# Supplementary material for: Detecting fatigue in multiple sclerosis through automatic speech analysis
Source: Front Hum Neurosci. 2024 Sep 13;18:1449388. doi: 10.3389/fnhum.2024.1449388 (PMC11427396; doi:10.3389/fnhum.2024.1449388)
Supplement: Supplementary file 1 [file Table_1.DOCX]

Supplementary Material

**Supplementary Table 1**

*Correlations between fatigue and other symptoms. Corrected for EDSS scores.*

|  | **Coefficient** | ***p-value*** | **Effect Size** | ***Adjusted p-value*** |
| --- | --- | --- | --- | --- |
|  | **FSMC - Total Score** | | | |
| Fatigue T-Score | 0.703 | 0.0 | 1.975 | 0.0 |
| HADS-Depression | 0.61 | 0.0 | 1.541 | 0.0 |
| Sleep T-Score | 0.583 | 0.0 | 1.436 | 0.0 |
| Stigma T-Score | 0.567 | 0.0 | 1.378 | 0.0 |
| Depression T-Score | 0.555 | 0.0 | 1.335 | 0.0 |
| Anxiety T-Score | 0.542 | 0.0 | 1.289 | 0.0 |
| HADS-Angst | 0.522 | 0.0 | 1.225 | 0.0 |
| EDSS Step | 0.452 | 0.0 | 1.014 | 0.0 |
| Lower Extremity T-Score | -0.395 | 0.0 | -0.86 | 0.0 |
| Upper Extremity T-Score | -0.478 | 0.0 | -1.087 | 0.0 |
| Satisfaction With Social Roles T-Score | -0.523 | 0.0 | -1.226 | 0.0 |
| Ability To Participate In Social Roles T-Score | -0.546 | 0.0 | -1.305 | 0.0 |
| Cognitive Function T-Score | -0.663 | 0.0 | -1.769 | 0.0 |
| SDMT Total Number Correct | -0.184 | 0.029 | -0.375 | 0.039 |
| Executive Function | -0.16 | 0.057 | -0.325 | 0.073 |
| Cognition Score | -0.136 | 0.109 | -0.274 | 0.129 |
| Memory Function | -0.099 | 0.243 | -0.199 | 0.272 |
| Processing Speed | -0.085 | 0.315 | -0.171 | 0.333 |
| Walk Duration | 0.059 | 0.489 | 0.119 | 0.489 |
|  | **FSMC - Motor Score** | | | |
| Fatigue T-Score | 0.658 | 0.0 | 1.746 | 0.0 |
| HADS-Depression | 0.582 | 0.0 | 1.433 | 0.0 |
| Stigma T-Score | 0.545 | 0.0 | 1.3 | 0.0 |
| Sleep T-Score | 0.543 | 0.0 | 1.293 | 0.0 |
| Depression T-Score | 0.541 | 0.0 | 1.285 | 0.0 |
| EDSS Step | 0.529 | 0.0 | 1.245 | 0.0 |
| HADS-Angst | 0.499 | 0.0 | 1.153 | 0.0 |
| Anxiety T-Score | 0.485 | 0.0 | 1.11 | 0.0 |
| Lower Extremity T-Score | -0.455 | 0.0 | -1.021 | 0.0 |
| Upper Extremity T-Score | -0.477 | 0.0 | -1.086 | 0.0 |
| Satisfaction With Social Roles T-Score | -0.517 | 0.0 | -1.209 | 0.0 |
| Ability To Participate In Social Roles T-Score | -0.535 | 0.0 | -1.265 | 0.0 |
| Cognitive Function T-Score | -0.583 | 0.0 | -1.437 | 0.0 |
| SDMT Total Number Correct | -0.174 | 0.039 | -0.353 | 0.053 |
| Executive Function | -0.155 | 0.067 | -0.313 | 0.085 |
| Cognition Score | -0.123 | 0.146 | -0.248 | 0.173 |
| Memory Function | -0.094 | 0.265 | -0.19 | 0.297 |
| Processing Speed | -0.057 | 0.504 | -0.114 | 0.528 |
| Walk Duration | 0.054 | 0.528 | 0.108 | 0.528 |
|  | **FSMC - Cognitive Score** | | | |
| Fatigue T-Score | 0.673 | 0.0 | 1.821 | 0.0 |
| HADS-Depression | 0.575 | 0.0 | 1.406 | 0.0 |
| Sleep T-Score | 0.573 | 0.0 | 1.399 | 0.0 |
| Anxiety T-Score | 0.57 | 0.0 | 1.388 | 0.0 |
| Depression T-Score | 0.526 | 0.0 | 1.235 | 0.0 |
| HADS-Angst | 0.499 | 0.0 | 1.152 | 0.0 |
| Stigma T-Score | 0.494 | 0.0 | 1.136 | 0.0 |
| EDSS Step | 0.32 | 0.0 | 0.675 | 0.0 |
| Upper Extremity T-Score | -0.393 | 0.0 | -0.854 | 0.0 |
| Satisfaction With Social Roles T-Score | -0.506 | 0.0 | -1.172 | 0.0 |
| Ability To Participate In Social Roles T-Score | -0.507 | 0.0 | -1.176 | 0.0 |
| Cognitive Function T-Score | -0.7 | 0.0 | -1.96 | 0.0 |
| Lower Extremity T-Score | -0.249 | 0.003 | -0.515 | 0.004 |
| Executive function | -0.127 | 0.132 | -0.257 | 0.179 |
| SDMT Total Number Correct | -0.115 | 0.176 | -0.231 | 0.223 |
| Cognition Score | -0.088 | 0.302 | -0.176 | 0.358 |
| Processing Speed | -0.071 | 0.406 | -0.141 | 0.454 |
| Walk Duration | -0.05 | 0.563 | -0.099 | 0.594 |
| Memory Function | -0.019 | 0.819 | -0.039 | 0.819 |

## FSMC: Fatigue Scale for Motor and Cognition. SDMT: Symbol Digit Modalities Test. HADS:  Hospital Anxiety and Depression Scale. EDSS: Expanded Disease Disability Status Scale. T-scores derived from the Neuro-QoL questionnaire.

**Supplementary Table 2**

*Description of features that were selected across all fold of the model’s cross validation*

| **Feature** | **Description** |
| --- | --- |
| Adjective rate | Proportion of adjectives across sentences. |
| Adverb rate | Proportion of adverbs across sentences. |
| Average MFCC (4) | Average Mel-Frequency Cepstral Coefficient. Provides particular information about the speech signal's spectral shape. |
| Brunet’s index | Brunet's statistic, a measure for lexical richness that is insensitive to the length of text. |
| Concept density | The ratio of total named concepts and total token count. |
| Conjunction rate | Proportion of conjunction across sentences. |
| F1 relative energy (SD) | Standard deviation of amplitudes of the spectral envelope at F1 in relation to the amplitude of the spectral F0 peak. |
| F3 frequency (mean) | Mean center frequency of third formant. |
| F3 frequency (SD) | Standard deviation of center frequency of third formant. |
| Hammarberg index (mean) | Mean ratio of the strongest energy peak in the 0-2 kHz region to the strongest peak in the 2–5 kHz region. |
| Local absolute jitter | Jitter is a measure of random perturbation in signal periodicity. More representative when examining long vowels. This is the average absolute difference between consecutive periods, in seconds. |
| Local shimmer (dB) | This is the average absolute difference between the amplitudes of consecutive periods, divided by the average amplitude. |
| Noun rate | Proportion of nouns across sentences. |
| Number of pauses | Number of pauses between speech segments based on voiced intervals. |
| Pause duration (mean) | Mean duration of pauses. |
| Pause duration (sum) | Total pause duration. |
| Pause durations (SD) | Standard deviation of pause durations. |
| Pause rate | Total length of pauses divided by the total length of speech (including pauses). |
| Pitch (linear regression offset) | Refers to the intercept of the linear regression line fitted to the pitch contour of a speech sample. This metric provides insight into the baseline level of the pitch when other factors are controlled. |
| Pitch (mean) | Mean pitch. Perceived frequency of a sound, corresponding to the rate of vibrations of the sound wave. |
| Pitch (first percentile) | The value of the fundamental frequency at the first percentile of its distribution in the speech sample. This value represents the very low end of the pitch range, showing how low the pitch gets in the sample. |
| Spectral slope in the 0-500 Hz band (mean) | Mean linear regression slope of the logarithmic power spectrum within the 0-500 Hz band. |
| Speech ratio | Percentage of the speech signal that is actually speech. |
| Type token ratio | Type-to-token ratio (i.e., the ratio of unique tokens). |
| Utterance Durations (SD) | Standard deviation of utterance durations. |
| Utterance durations (sum) | Total duration of utterances. |
| Word frequency (mean) | Word frequencies across all words. |

## SD: standard deviation. dB: decibel.
